# The prevalence and co-occurrence of emulsifiers, thickeners and stabilizers in food products on the Norwegian market

**DOI:** 10.29219/fnr.v69.12986

**Published:** 2025-12-08

**Authors:** Åsne Skram Trømborg, Jon Olav Vik, Monica Hauger Carlsen, Harald Carlsen

**Affiliations:** 1 Faculty of Chemistry, Biotechnology and Food Science, Norwegian University of Life Sciences, Ås, Norway; 2 Faculty of Medicine, University of Oslo, Oslo, Norway

**Keywords:** food composition, ingredient analysis, food matrix, ultra-processed foods, additive combinations

## Abstract

**Background:**

As the intake of food that has undergone substantial processing increases, the intake of common additives like Emulsifiers, Thickeners, and Stabilizers (ETSs) also increases. Several ETSs have drawn attention due to potential negative health effects.

**Objective:**

This study aimed to map the prevalence and co-occurrence patterns of ETSs across food products on the Norwegian market, examining their distribution across different food groups and products with varying nutritional profiles, including those classified as hyper-palatable.

**Design:**

Food product information, including ingredient lists and nutritional information, was obtained from two online databases used by major grocery chains, thus representative of the Norwegian market and analyzed for presence or absence of ETSs.

**Results:**

Thirty-two percent of food products contained at least one ETS, 60% of these contained more than one ETS, and many ETSs were found more frequently in combinations with others than individually. ETSs were present in a broad range of food products, with the highest percentage seen in the food groups Bakery, Cakes & Pastries, and Desserts & Ice cream. Mono- and diglycerides of fatty acids (E471) and diphosphates (E450) were the most used food additives. Fifty-eight percent of the food products analyzed were classified as hyper-palatable, though relationships with ETSs were variable across food groups.

**Conclusion:**

This is the first comprehensive analysis of its kind, providing a basemap for future research on the potential health effects of modern food products, particularly considering the limited understanding of how multiple ETSs may interact within biological systems.

## Graphical Abstract

**Figure:**
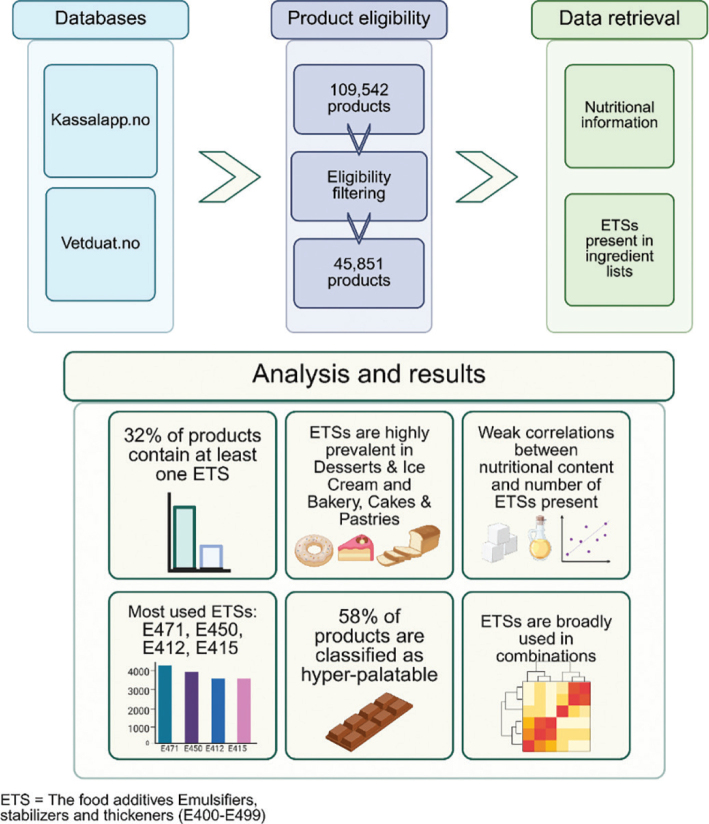

## Popular scientific summary

- Dietary intake of Emulsifiers, Thickeners and Stabilizers (ETS) has been implicated in potential health issues.
- We present the first comprehensive overview of the prevalence and co-occurrence of ETSs across food groups, finding that 32% of products contain ETSs. Where ETSs occur, they tend to occur in combination, which calls for more multi-factor studies.
- This study provides a basemap for future research on health implications of a modern Western diet.

Modern food industry uses additives to increase shelf-life, enhance food safety, preserve or change colors, increase nutritional value, and modify flavor and texture (1). Food processing technologies and additives developed throughout the 20th century have enabled large-scale production, lower production and purchase costs, and availability of ready-to-eat products (1–3). Today, the typical Western style diet includes an increasing number of foods that have undergone substantial industrial processing, and with that an abundance of food additives. Estimates suggest that intakes of energy (E%) from these food types range from 14E% in Italy and Romania (4), 48E% in Norway (5) to 58E% in the US (4, 6–8). Intakes of highly processed foods have been associated with negative health outcomes, such as weight gain in cohort studies using 24-h dietary recalls (9–13) and randomized controlled trials (14), as well as non-communicable lifestyle diseases in meta-analyses synthesizing findings from studies using various dietary assessment methods, including food frequency questionnaires (FFQs) (15, 16). While meta-analyses include studies with different dietary methods, it is important to consider that FFQs may introduce measurement errors when estimating intakes of highly processed foods.

Further, the mechanisms behind these associations are not completely understood. Highly processed foods often have a less ideal nutrient profile compared to minimally processed foods, being for example energy dense, high in sugar and sodium, low in dietary fiber and high in saturated fat (7, 8, 17–19). Although there is consensus that the abovementioned attributes contribute to poorer health outcomes, food additives, food texture, and degree of hyper-palatability are also suggested to play a role in mediating negative health effects of highly processed foods (20–23).

Food additives encompass a wide range of substances used, among other things, to enhance flavor, appearance, preservation, and texture. Among these, emulsifiers, thickeners, and stabilizers (ETSs) are particularly relevant due to their role in modifying texture and viscosity – properties that influence both sensory appeal and potentially eating behavior. Their widespread use in processed foods (24, 25) combined with studies suggesting negative health outcomes make ETSs a relevant focus for investigation.

Approximately 60 ETSs are approved for use in Europe, according to Regulation (EC) No 1333/2008 Annex II (26), and are grouped together due to their functional properties, presented in Annex I. While they share many functional properties, their origins varies widely. Many are naturally derived from plant, microbial, or marine sources, while others are synthetic or chemically modified (27). These additives are classified as ETSs – each with distinct functions in food processing. Emulsifiers are used to homogenize immiscible phases, such as fat and water, creating stable mixtures in products like sauces and spreads. Thickeners modify the texture by increasing the viscosity of foods, contributing to a desirable mouthfeel and consistency. Stabilizers help maintain the uniform dispersion of ingredients over time, preserving the consistency, color, and flavor of products such as beverages and dressings. Some ETSs also contribute to gel formation and hence improved integrity of certain foods.

Importantly, the physical properties imparted by ETSs – such as texture and viscosity – may influence consumption patterns and digestive outcomes (28, 29). In addition, approximately 20 of these additives are unlikely to be absorbed in the upper gastrointestinal tract and instead reach the colon undigested (30–44), as presented in Table 1. Since these additives are complex carbohydrates with structures similar to dietary fibers, concerns have been raised that they adversely affect gut microbiota composition (45–54). As an example, Chassaing et al. demonstrated that a daily intake of 15 grams of carboxymethylcellulose (E466) altered gut microbiota composition in healthy adults that led to depletion of health promoting bacterial metabolites (55).

**Table 1:** Additives in class E400–499, Emulsifiers, Thickeners and Stabilizers (ETSs), and their suggested fate in the human gastrointestinal system

Non-absorbed ETSs		Absorbed ETSs	
E-number	Name or group	E-number	Name or group
**E400–E404**	Alginic acids and its salts (30)	E420–E422	Sugar alcohols
**E405**	Propane-1,2-diol alginate (hydrolyzed to propane-1,2diol and alginate (31))	E431–E436	Polyoxyethene compounds
**E406**	Agar (32)	E442–E445	Non-polysaccharide emulsifiers from natural sources
**E407**	Carrageenan (33)	E450–E452	Phosphates
**E410–E427**	Gums (34–42)	E470–E477	Derivatives of fatty acids
**E440**	Pectins (43)	E481–E482	Lactylates
**E460–E469**	Celluloses (44)	E491–E495	Partial esters of sorbitol and its anhydrides
		E456	Potassium polyaspartate
		E459	Beta-cyclodextrin
		E479b	Thermally oxidized soybean oil
		E483	Stearyl tartrate
		E499	Stigmasterol-rich plant sterols

Animal studies have shown that carrageenan (E407), carboxymethyl cellulose (E466), and polysorbate 80 (E433) can worsen colitis symptoms through alterations of the gut microbiota (56–60) and contribute to metabolic syndrome (61, 62). However, these findings may have limited relevance to humans due to high doses and non-physiological administration. Additionally, most animal research investigates single ETSs, ignoring possible interactions with other ETSs or the food matrix.

Only a few human studies have been carried out. In addition to the study by Chassaing et al. (55), observational data from the NutriNet-Santé cohort associated intake of celluloses (E460–E469) and fatty acid derivatives (E471, E472) with cardiovascular disease (63) and carrageenan (E407), guar gum (E412), gum arabic (E414), and xanthan gum (E415) with type II diabetes (64).

Food additives are consumed alongside other nutrients and compounds, not in isolation. Additionally, food characteristics like texture, viscosity, palatability, and energy density influence intake and eating behavior (20, 28, 29, 65). To better understand how complex food matrices affect health, we must move beyond studying single components and consider the broader food context. The first step is to map where and how additive groups like ETSs are used across food categories, and how they relate to nutritional and sensory profiles. While such mapping has been done in France (excluding nutrition) (66) and for emulsifiers in UK ultra-processed foods (UPFs) (24), it remains important to explore whether similar patterns exist in other countries, such as Norway.

This study aimed to systematically map the presence of the E400–E499 additives, the ETSs, in food products on the Norwegian market, examining their distribution across different food groups and products with varying nutritional profiles. Specifically, we charted the extent and patterns of co-occurrence of these ETSs in foods with differing types of hyper- palatability, and energy, fat, carbohydrate and sodium content. Understanding where these ETSs are commonly found and their association with nutritional characteristics provides a foundation for future studies on their potential health implications. We hypothesized that ETSs are not randomly distributed across food products, but rather tend to co-occur in specific food groups and in products with hyper-palatable characteristics and higher energy, fat, carbohydrate, or sodium content.

## Methods

### Food products and eligibility

Food product information was obtained from the two food information databases, *Vetduat.no* and *Kassalapp*^®^, on October 21^st^, 2024. *Vetduat.no* is owned by Tradesolution AS, and contains information from 1497 companies, as of May 22^nd^, 2024. *Kassalapp*^®^, owned by Liseth Solutions AS, contains product label information, provided by groceries stores in Norway, obtained through information available at the grocery store’s websites. R, *version 4.4.2*, through RStudio, *version 2024.12.1*, was used for all processing of data. The package *tidyverse, version 2.0.0* (67) were used for data processing, unless otherwise specified. The compiled food information included ingredient lists and compulsory nutrient labelling (energy, carbohydrates, sugar, salt, fat, saturated fatty acids, and protein).

**Fig. 1:**
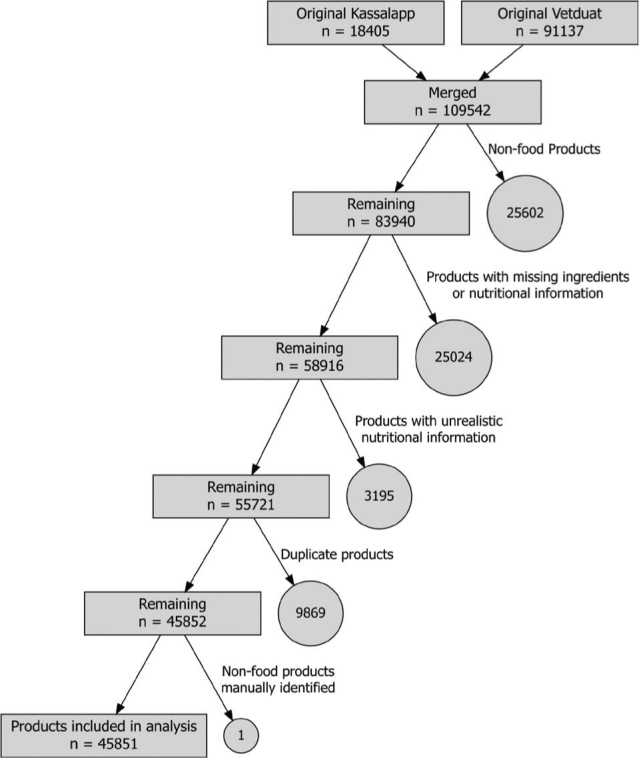
Inclusion chart of food products to be used in downstream analysis

Products that did not meet eligibility were removed. These included non-food products, products without ingredient lists, food products with unrealistic nutritional information (greater amount of nutrients per 100 g than 100 g, more sugar than total carbohydrates, more saturated fat than total fat, no energy, or more than 4,200 kJ per 100 g, or energy ratio between kcal and kJ not being between 3.984 and 4.384), and duplicate products based on name or Global Trade Item Number (GTIN). For products with low energy content (kJ < 10 and kcal < 2) the energy ratio filtering step was not applied. Of a total of 109,542 products obtained from the two databases, 45,851 unique products met eligibility and were used for further analysis. See the flow chart for inclusion of products in Fig. 1.

### Identification of ETSs

Ingredient lists were searched for ETSs based on E-number, plaintext names, and synonyms, accounting for common misspellings, using regular expressions such as ‘Carrageenan E407 = ((c|k)ar*age*nan | E[ -]?407)’. For ETSs that were listed several times in the same product (such as in separate ingredient lists for two components in the same product), the specific ETS was counted once. Sub-classes of ETSs, such as E472a and E472b, were not distinguished individually but were included in their main class.

### Grouping of food products

Food products in both databases were originally assigned to food groups, with slight differences in classification. These groupings were regrouped to match the food categories presented in Table 2. Manual grouping was performed only for products that lacked a predefined food group.

**Table 2:** Food groups used in this study

Food group
Desserts & Ice Creams
Beverages
Fish & Shellfish (and products thereof)
Meat & Poultry (and products thereof)
Fats, Margarine & Spreads
Premade Food & Dinner Kits
Snacks, Chocolate & Sweets
Bakery, Cakes & Pastries
Eggs & Dairy Products
Grains, Baking Mixes & Cereals
Baking Condiments
Sauces, Dressings & Other Dinner Condiments
Processed Fruits & Vegetables
Fruit, Vegetables & Legumes
Infant Food
Others

### Nutritional information and palatability

Norway requires declaration of nutritional information including energy, fat, saturated fatty acids, carbohydrates, sugars, protein, and salt. Sodium content was estimated as 40% of the declared salt content. This nutritional information was used for further analysis.

We used Fazzino et al.’s operational definition of hyper-palatable foods as those meeting one or more of three criteria: 1) fat and sodium content (> 5E% from fat & ≥ 0.30% sodium by weight); 2) fat and sugars content (>20E% from fat & >20E% from sugar); 3) carbohydrates (without sugar) and sodium content (>40E% from carbohydrates & ≥ 0.20% sodium by weight) (68). Thus, we classified products into their hyper-palatability group, or in the Fits >1 group for those who met more than one criterion. Beverages were excluded from this classification. The number of products included in the palatability analysis was 41,899.

We estimated the percentage energy contribution (E%) from fat and carbohydrates, to be used in the palatability analysis, in each food item as follows: the amount of total fat, in gram per 100 g was multiplied by 37 kJ/g, and the carbohydrate and sugar contents were multiplied by 17 kJ/g, as specified in Regulation (EU) No 1169/2011 on the provision of food information to consumers (69). To obtain E%, energy from fat and carbohydrates in each food item was divided by the declared total energy content, including all energy-providing nutrients (in kJ/100 g), and multiplied by 100.

The final dataset included the food products names and identifiers, their nutritional information, assigned food group, presence of ETSs (E400–E499), and hyper-palatability status based on the defined conditions.

### Presence of ETSs and distribution of ETS counts

To determine the prevalence of food products containing at least one ETS, each product was first classified based on the binary presence or absence of an ETS. Products containing one or more ETSs were then further categorized by the number of ETSs present. The categories included products with 1 to 10+ ETSs. This classification allowed for the calculation of the proportion of food products containing none to 10+ ETSs, across all food products, within specific food groups, and within different classes of hyper-palatability. Additionally, ETSs believed to pass undigested to the large intestine were marked accordingly.

### Statistical analysis

The data were a near-complete list of food products available at one point in time. To interpret it statistically, we conceptualize a broader population of potential products that could have been marketed. Sample sizes were large enough to detect even very small departures from many null hypotheses, and p-values alone are therefore of limited interest. Instead, we emphasize descriptive statistics and visualizations (box plots, bar charts, pie charts), and supplement these with interval estimation of fold enrichment (see further in the text). The main limitation is the single time-point, which precludes inference about temporal trends.

Unless otherwise specified, all figures were created using *ggplot2* in *tidyverse.* Box plots were used to visualize the nutrient content of food products with differing numbers of ETSs present. To test the association between nutritional content and number of ETSs, the Spearman rank correlation test was performed.

To visualize the distribution of ETSs across food groups, we generated a heatmap of the percentage of food products in each group containing specific ETSs. A binary presence–absence matrix was constructed and visualized using the package *pheatmap, version 1.2* (70). A co-occurrence plot was created using the package *ComplexUpset* (71). Briefly, the list of ETSs was split and transformed into a binary matrix, where presence was encoded as 1. An UpSet plot was generated, excluding combinations found in fewer than 50 products.

Within each food group, associations among ETS combinations were quantified by ‘fold enrichment’, defined as the observed-to-expected ratio of the number of products having a given combination (say, E1 & E2 & E4) to the number expected under the null hypothesis that the ETSs occur independently, conditional on the marginal probabilities (so expected = N × P(E1) × P(E2) × P(E4) in the example). We extended the one-sided *SuperExactTest* method (72) to get two-sided p-values so we could test for both over- and under-representation, using Blaker’s improved ‘exact’ method (73). To focus on a tractable number of combinations which affected a non-negligible number of food products, we first lumped groups of additives with very similar properties (E40x = E440–444, E42x = E420–E422, E45x = E450–E452, E47x = E470–E477), then included only ETSs present in at least 1% of products in the food category, and tested only combinations with an expected product count of 10 or more. The null model (a multiset hypergeometric under independence) has no free parameters to describe a degree of deviation from null, so conventional confidence intervals cannot be defined. Instead, we computed bootstrap percentile intervals of log fold enrichment (using the core R package *boot*) to focus on deviations from null which were both statistically significant and substantially interesting.

## Results

### Presence of ETSs across all food products

**Fig. 2:**
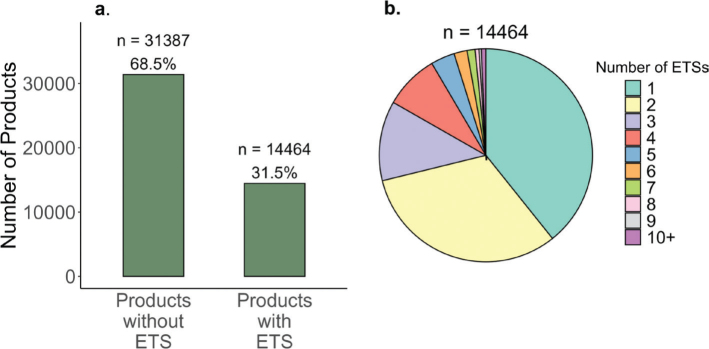
Distribution of food products with and without Emulsifier, thickener or stabilizer (ETS). (a) The number (*n*) and proportions (%) of products containing none or at least one ETS. (b) Distribution of the number of products (n) with one and up to 9 and 10 or more (10+) ETSs among products.

A total of 45,851 food products were analyzed. As shown in Fig. 2, panel A, 68.5% of products did not contain any ETSs, while 31.5% (14,464 products) contained at least one ETS. The pie chart in panel B further shows that among these, 40% contained one ETS, 32% contained two ETSs, 12% contained three ETSs, and 16% contained four or more ETSs.

### Presence of ETSs in different food groups

**Fig. 3:**
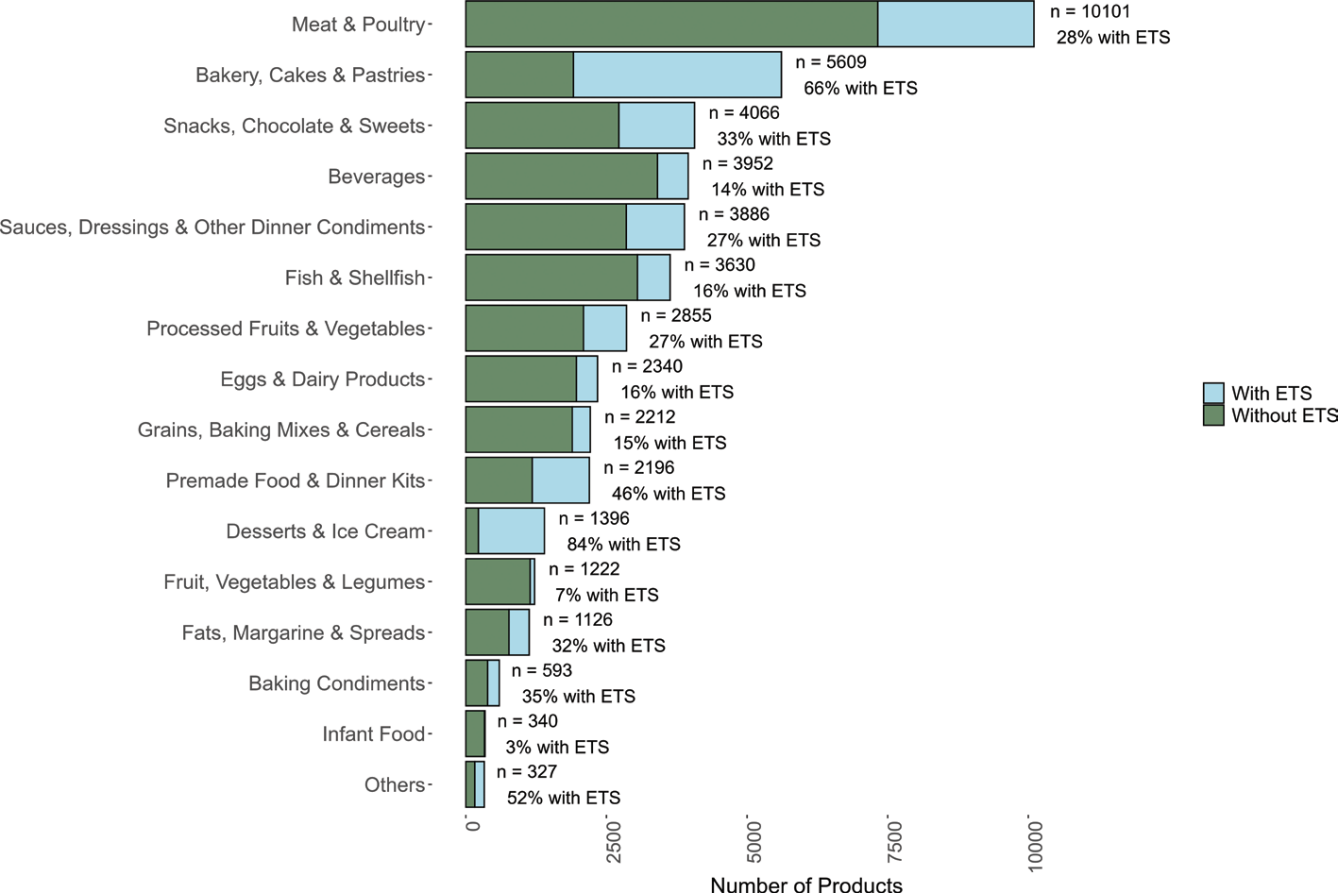
Number of products (*n*), and the proportion (%) of products in each food group containing at least one ETS indicated by blue color.

Figure 3 shows the number of products in each food group and the proportion of products containing at least one ETS. The largest food group in our dataset was Meat & Poultry, of which 22% (10,101 products) belonged to this group. Twenty-eight percent (2,778 products) of these contained at least one ETS, shown in Fig. 3. The food category with the highest proportion of products containing ETSs was Desserts & Ice Cream, where 84% of the 1,396 products included at least one ETS. This was followed by Bakery, Cakes & Pastries, in which 66% of the 5,609 products contained at least one ETS. In contrast, Infant Food exhibited the lowest proportion, with only 3% (10 products) of the 340 total food products containing one ETS.

**Fig. 4:**
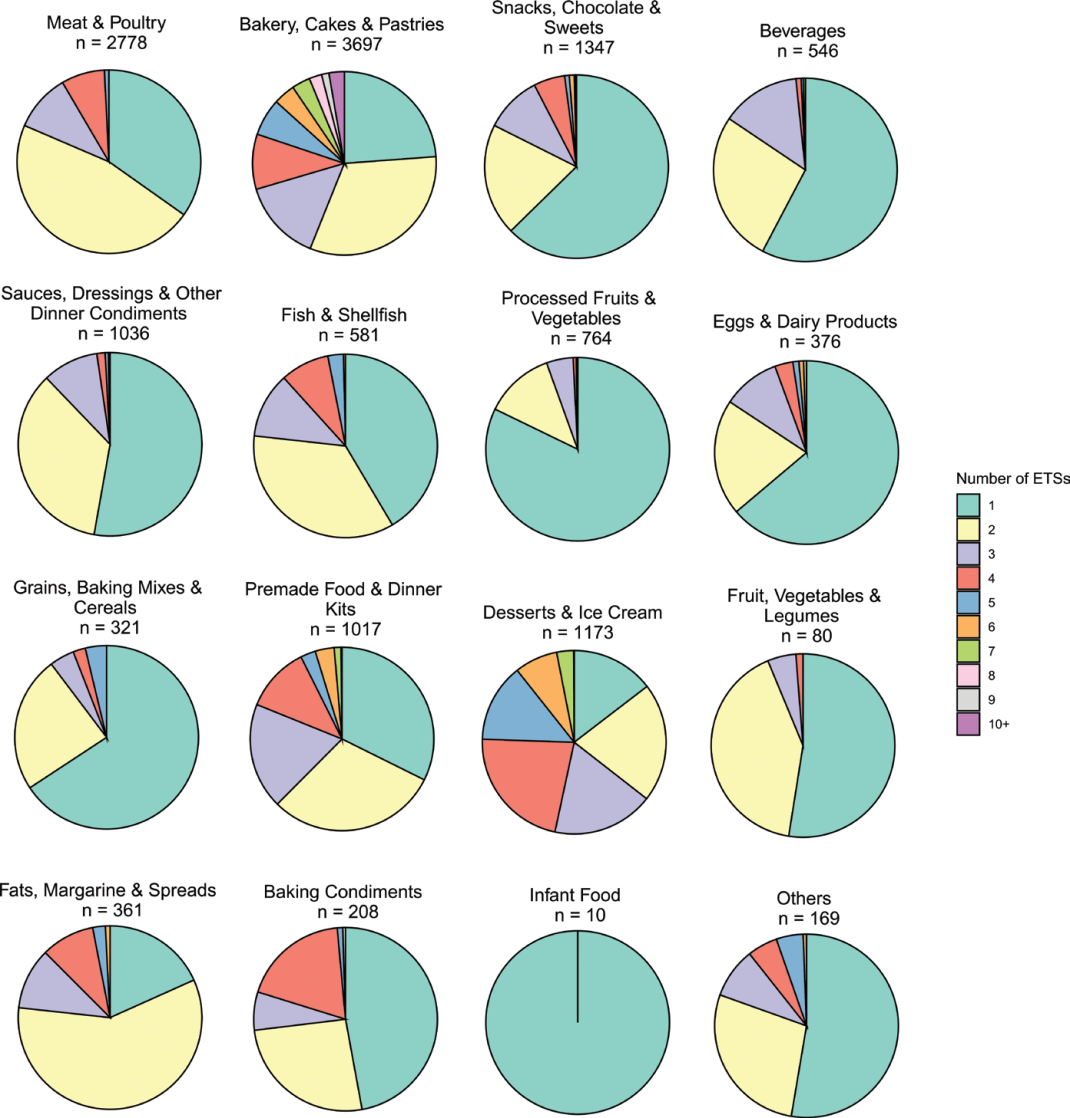
Distribution of the number of products (n) with one and up to 9 and 10 or more (10+) ETSs among products in the different food groups.

Figure 4 presents a series of pie charts, each representing a food group, showing the distribution of products according to the number of ETSs they contain. As shown, across most food groups and among products containing ETSs, the presence of exactly one ETS is more common than any other specific count. Exceptions were Dessert & Ice cream, where four ETSs was most common, and Fats, Margarine & Spreads, Meat & Poultry, and Bakery, Cakes & Pastries, where two ETSs were most prevalent. Infant food products never contained more than one ETS.

### Hyper-palatable foods and the presence of ETSs

**Fig. 5:**
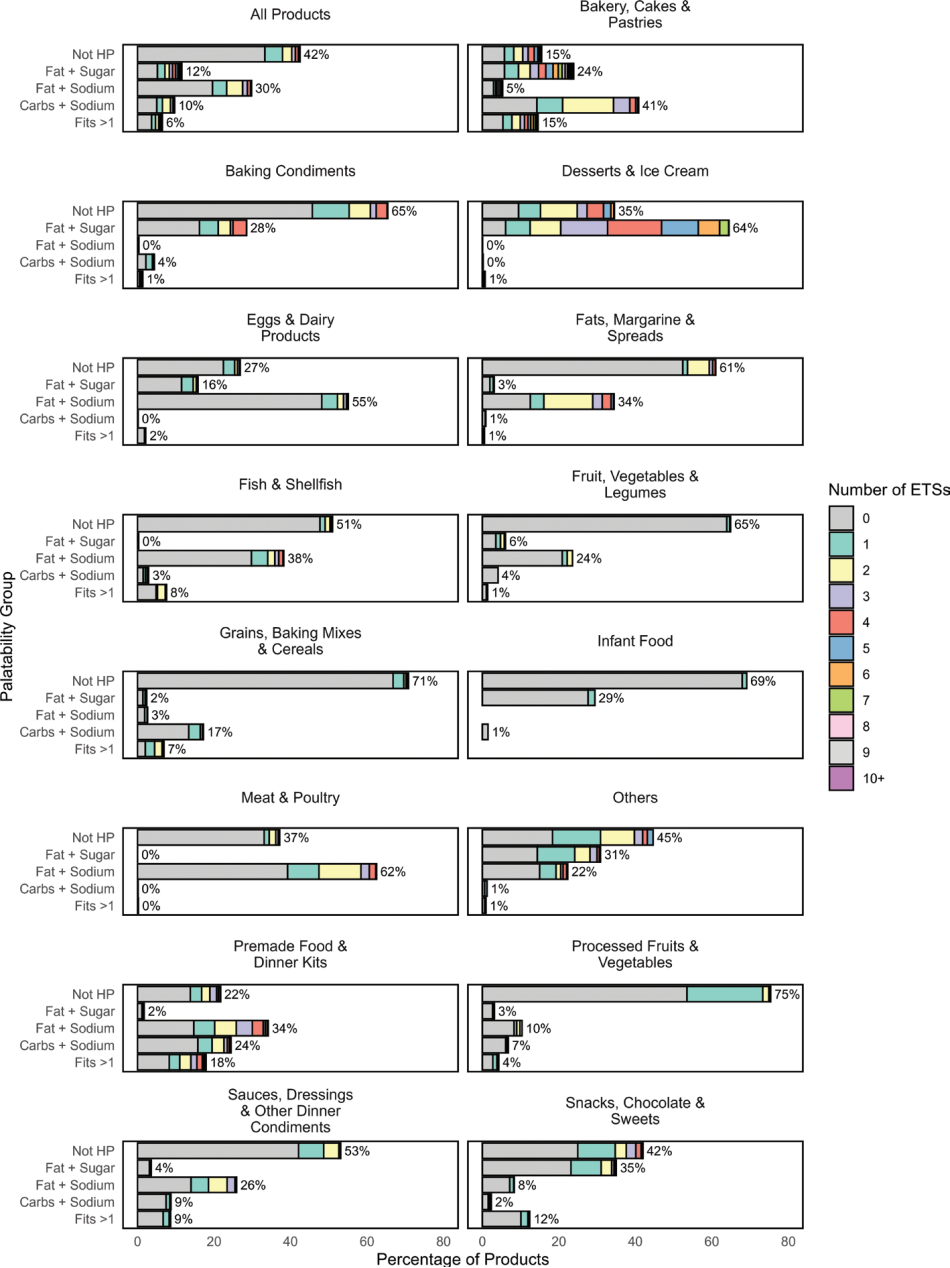
Distribution of food products by hyper-palatability classification – those not meeting any criteria (Not HP), those meeting specific criteria (Fat + Sugar, Fat + Sodium, or Carbohydrate + Sodium), and those meeting more than one criterion (Fits >1) – alongside the distribution of products containing 0–9 and 10 or more (10+) ETSs, shown for all products combined and across different food groups.

Figure 5 presents the distribution of food products by hyper-palatability classification across all products and food groups. The figure uses grouped bar charts to show the proportion of products that did not meet any hyper-palatability criteria (Not HP), and those that met one of the three criteria: Fat + Sugar, Fat + Sodium, or Carbohydrate + Sodium, as well as those that met more than one criterion. Of the 41,899 products included in the palatability analysis, 42% were not hyper-palatable. In total, 12% matched the Fat + Sugar criterion, 30% the Fat + Sodium criterion and 10% the Carbohydrate + Sodium criterion. Six percent matched more than one criterion. The food group Bakery, Cakes & Pastries had the highest proportion of products being hyper-palatable: 24% Fat + Sugar; 41% Carbohydrate + Sodium; 15% fit more than one criterion. Only 15% of foods in this category were judged to be not hyper-palatable. Desserts & Ice Cream had the greatest proportion of products being in one hyper-palatable group, with 64% of food products matching the Fat + Sugar criterion. Grains, Baking Mixes & Cereals, and Processed Fruits & Vegetables had the lowest proportion of hyper-palatable food with 75% not complying with any of the criteria.

### Nutritional values and presence of ETSs

**Fig. 6:**
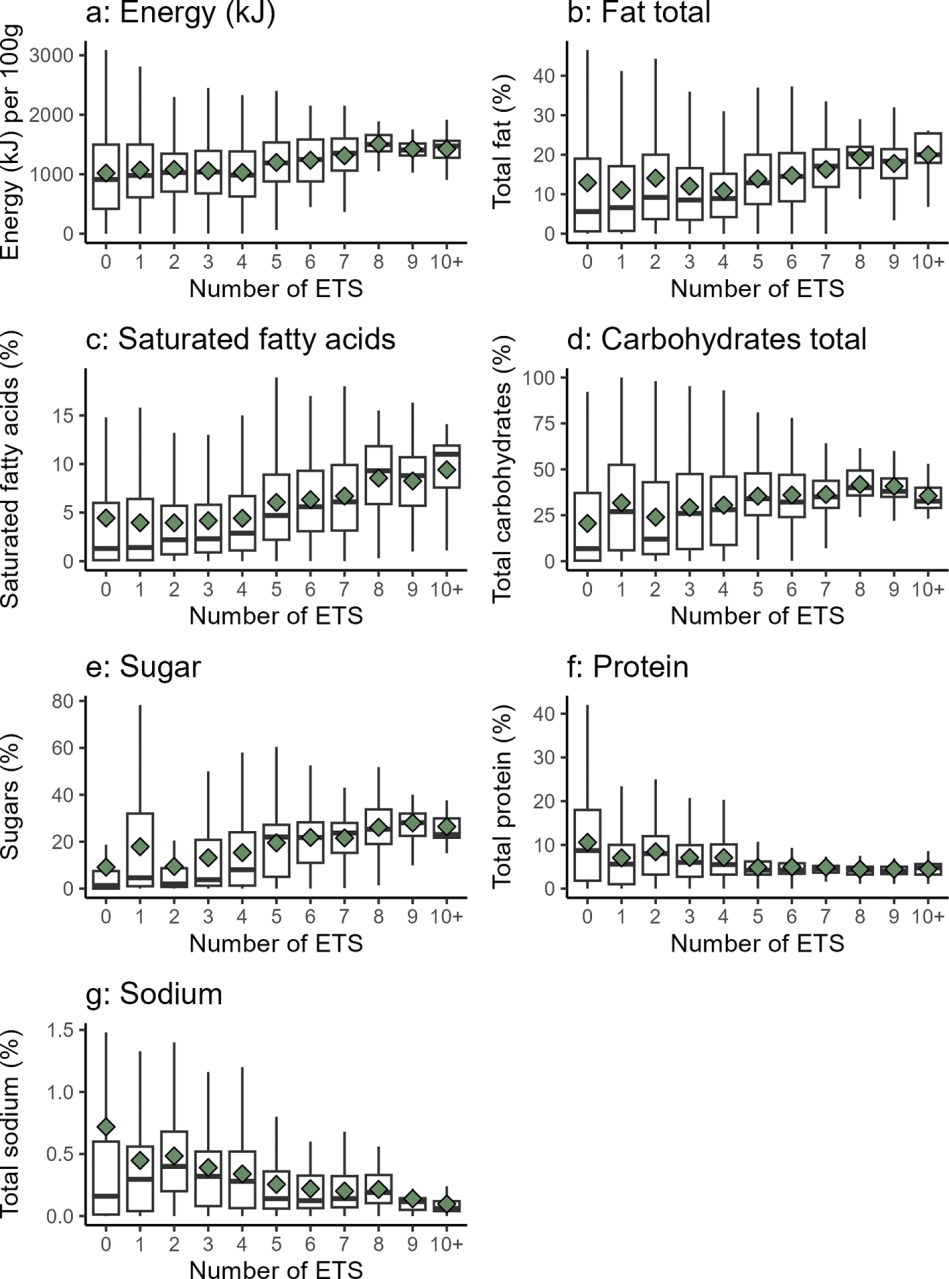
Box plots illustrating the distribution of various nutritional values (Y-axis) across food products grouped by the number of different ETSs present (X-axis), ranging from 0 to 10 or more (10+). Median values are shown as black lines, and means are indicated by green diamonds. Boxes represent the interquartile range (25th to 75th percentiles), and whiskers extend to the minimum and maximum values, excluding outliers.

Figure 6 presents a series of box plots showing the distribution of nutritional values across products grouped by the number of ETSs they contain (from 0 to 10 or more). Each subplot displays one nutrient attribute: energy (kJ), total fat, saturated fatty acids, total carbohydrates, sugars, protein, and sodium. Median values are indicated by black lines, and means by green diamonds. The number of ETSs showed weak but statistically significant (*p* < 0.0001) positive correlations with energy, total carbohydrates and sugars, total fat, saturated fatty acids, and total sodium. In contrast, protein content was weakly, though significantly (*p* < 0.0001) negatively correlated with the number of ETSs, presented in Table 3. These patterns may suggest potential trends, although the associations are weak.

**Table 3:** Spearman rank correlation between number of additives (0–10+) and nutritional content

Nutrient	Spearman correlation
Sugars (%)	0.242**
Total carbohydrates (%)	0.228**
Total protein (%)	–0.128**
Saturated fatty acids (%)	0.120**
Total fat (%)	0.097**
Energy (kJ) per 100 g	0.093**
Total sodium (%)	0.098**

** *p* < 0.0001.

### Count of each ETS and their presence in food groups

**Fig. 7:**
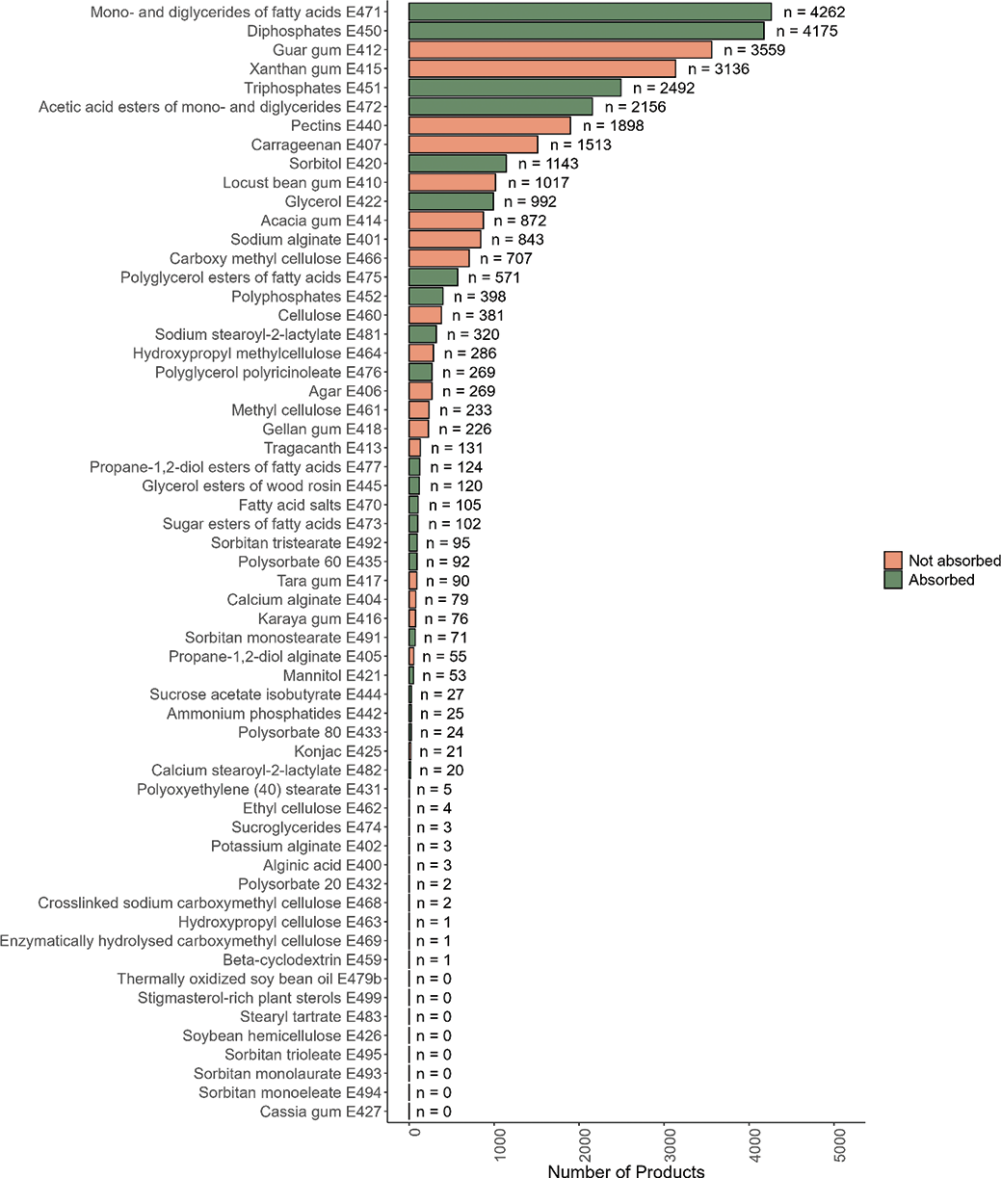
The number of products in which each ETS is found across food products. ETSs that are thought to reach the colon unabsorbed are marked in pink, while ETSs that are absorbed intact or partly degraded are marked in green.

Figure 7 shows the number of food products containing each ETS. The most frequently occurring ETS in the present analysis was mono- and diglycerides of fatty acids (E471), which was found in 4,262 food products, followed by diphosphates (E450), found in 4,175 food products. Among ETSs that reach the colon undigested, guar gum (E412) and xanthan gum (E415) were found most frequently, with 3,559 and 3,136 products containing these ETSs, respectively. Of the 59 ETSs analyzed in this study, 31 ETSs were found in less than 100 products – representing 0.2% or fewer of all food products included in the analyses. The following ETSs were not found in any food products in our dataset: stigmasterol-rich plant sterols (E499), stearyl tartrate (E483), soybean hemicellulose (E426), sorbitan trioleate (E495), sorbitan monolaurate (E493), sorbitan monoeleate (E494), cassia gum (E427), and thermally oxidized soybean oil (E479b).

**Fig. 8:**
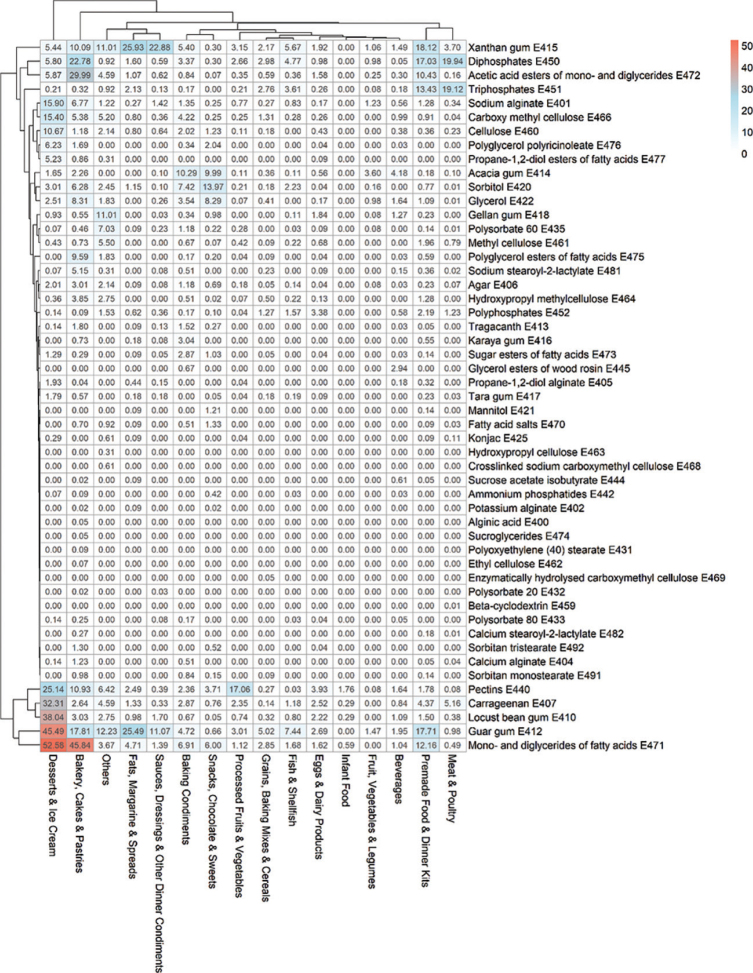
Heatmap showing the percentage of food products within each food group that contain specific ETSs. Only ETSs present in the dataset are included. Color intensity ranges from blue (lowest percentage) to red (highest percentage), indicating the proportion of products containing each ETS. The figure was generated using the *pheatmap* package in R.

Figure 8 presents a heatmap showing the prevalence of each ETS across different food groups, allowing visual comparison of both frequently and rarely used additives. The distribution of ETSs across the different food groups showed great variation in the prevalence of the different ETSs. The highest prevalence of ETS in a food group was mono- and diglycerides of fatty acids (E471) which was found in 53% of the products in Dessert & Ice Cream, and in 46% products of Bakery, Cakes & Pastries. Our results also show that several food groups had a near absence of different ETSs, and vice versa, that is that also several ETSs had a low prevalence across all food groups.

### ETS co-occurrence

**Fig. 9:**
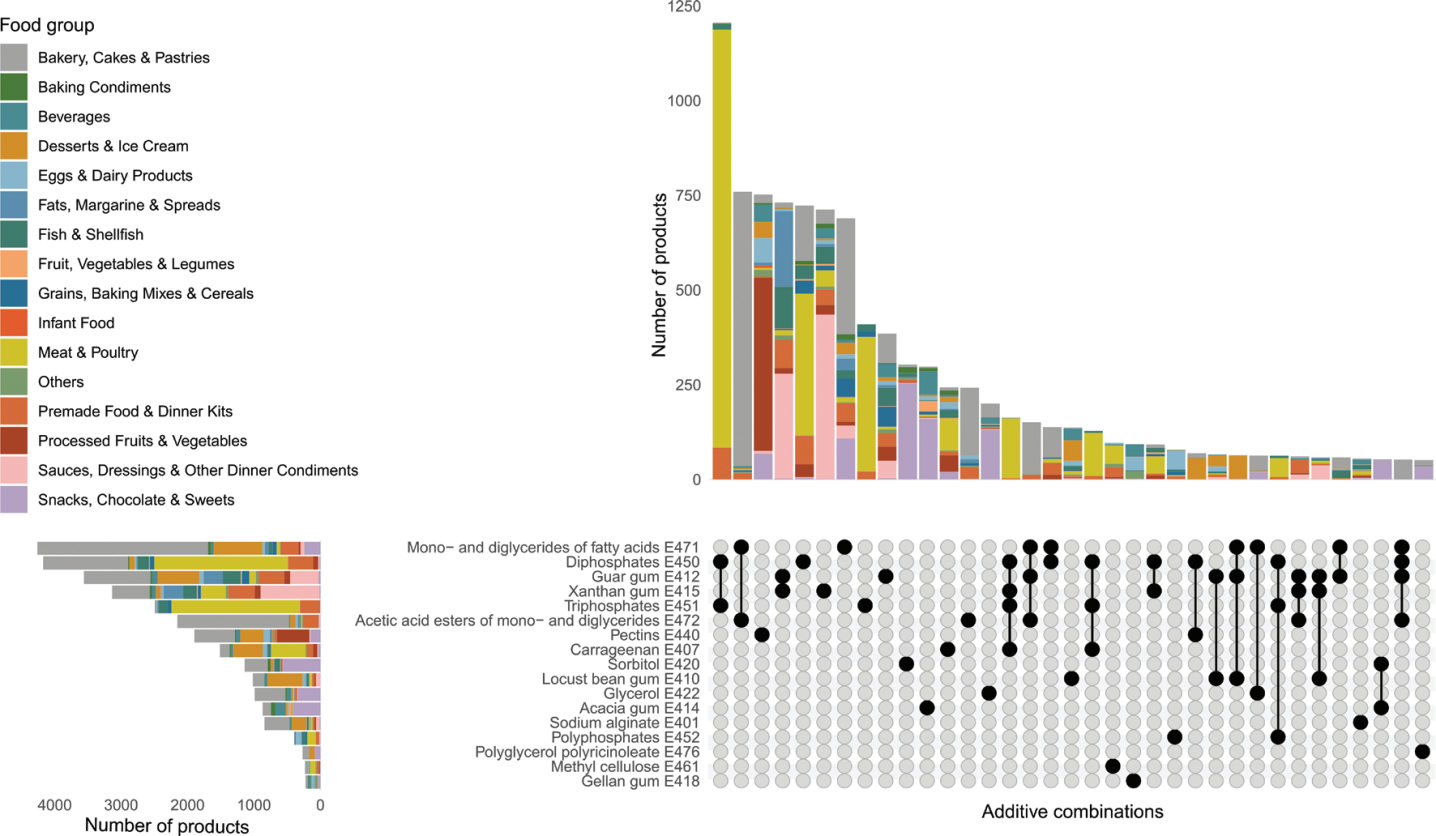
Distribution of additive combinations across food groups. The bar chart (top right) shows the number of food products containing specific combinations of ETSs, with colors indicating the associated food group. The dot plot (bottom right) visualizes which additives are included in each combination. The bar chart (bottom left) displays the frequency of individual additives across food groups. Only additive combinations present in at least 50 products are included.

Figure 9 shows ETS co-occurrence patterns across food groups. The top-right bar chart shows the number of products containing exact combinations of ETSs, color-coded by food group. The bottom-right matrix plot indicates which ETSs are included in each combination, while the bottom-left bar chart displays the frequency of individual ETSs across food groups. Only combinations found in at least 50 products are shown, emphasizing commonly co-occurring additives and their associated food categories. Many ETSs occurred most frequently together. Specifically, di- and triphosphates (E450 and E451) tended to co-occur, with Meat & Poultry products being the primary contributing food group. Similarly, guar gum (E412) and xanthan gum (E415) were commonly found together, particularly in Sauces, Dressings & Other dinner condiments, as well as in Fats, Margarine & Spreads. Further, mono- and diglycerides of fatty acids (E471) and acetic acid ester of mono- and diglycerides of fatty acids (E472) often appeared together in products classified as Bakery, Cakes & Pastries. In contrast, the most frequent ETS found alone was pectin (E440), with Processed Fruit & Vegetables being the primary food group containing pectin as the sole added ETS.

**Fig. 10:**
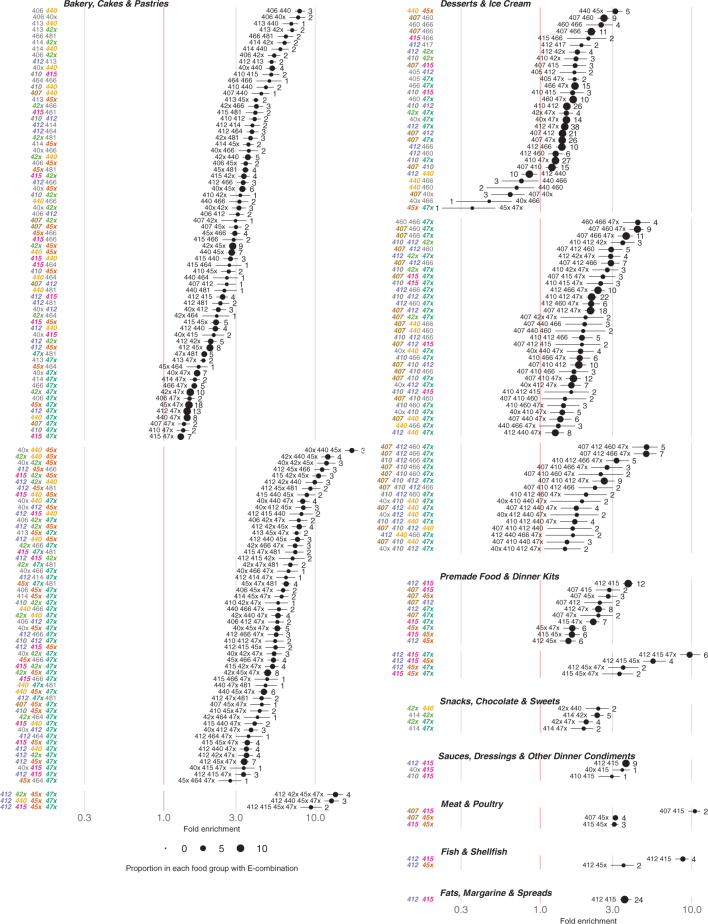
Fold enrichment of additive combinations (ranging from two to four additives, and present in at least 1% of their food group) within each food group, relative to expectations under the assumption of independence. Black dots indicate the median fold enrichment from bootstrap resampling, with horizontal lines representing the corresponding bootstrap confidence intervals. Dot size reflects the proportion of products in the food group that contain the given combination. For clarity we show only combinations of 2- 4 additives. Note that the combinations are “at least” (e.g. “412–415” means “E412, E415, and possibly others”), in contrast to the “exact” combinations used in Figure 9. Lumped additives: E40x=E400-404, E42x=E420-E422, E45x=E450-E452, E47x=E470-E477.

Figure 10 shows the fold enrichment of additive combinations that are over- or underrepresented compared to what would be expected under the assumption that such combinations occurred independently. The figure also shows the proportion of each combination within the food group, along with bootstrap-estimated confidence intervals.

The food groups Desserts & Ice Cream and Bakery, Cakes & Pastries exhibited the highest number of significantly enriched combinations and are the only groups with enriched combinations consisting of four additives. In Desserts & Ice Cream, the pairwise combination with the highest fold enrichment was pectin (E440) and phosphates (E450–E452), with a fold enrichment of approximately three. Among three-way combinations, cellulose (E460), carboxymethyl cellulose (E466), and fatty acid derivatives (E470–E477) showed the strongest enrichment. The four-way combination of carrageenan (E407), guar gum (E412), cellulose (E460), and fatty acid derivatives (E470–E477) in Desserts & Ice Cream had the highest fold enrichment overall for this food group, with a value of five. In the same food group, six pairwise combinations occurred significantly less frequently than expected based on the marginal probabilities of the individual additives. Notably, the combination of phosphates (E450–E452) and fatty acid derivatives (E470–E477) was significantly underrepresented.

In contrast, the food groups Meat & Poultry; Sauces, Dressings & Other Dinner Condiments; Fats, Margarine & Spreads; and Fish & Shellfish had only one, two, or three combinations enriched, all consisting of pairwise combinations. Notably, xanthan gum (E415) appeared in seven of the nine enriched pairs across these groups, spanning four unique combinations.

The pair guar gum (E412) and xanthan gum (E415) emerged as the most frequently enriched combination, appearing as the highest enriched pair in four out of the eight food groups included in this analysis, as well as being present in 24% of all products in Fats, Margarine, and Spreads, and 12% of Premade Food & Dinner Kits.

## Discussion

To the best of our knowledge, the present study is the first to systematically map the presence of additives within the class of ETS (E400–E499) in products available on the Norwegian market, with relevance for other countries where processed foods are prevalent. Additionally, it provides an analysis of their co-occurrences and distribution across food products with varying nutritional profiles.

In our dataset, about one third of the food products contained at least one ETS. Desserts & Ice Cream and Bakery, Cakes &Pastries had the highest proportions of ETSs. Over half of the products in our analyses were classified as hyper-palatable, but very weak correlations were found between the contents of nutrients (i.e., fat, carbohydrates, sodium, and protein) and number of ETSs present. Mono- and diglycerides of fatty acids (E471) were the most prevalent ETS, while over half of the ETSs were rarely found. Mono- and diglycerides of fatty acids (E471) were especially common in Desserts & Ice Cream and Bakery, Cakes & Pastries. Guar gum (E412) was found in almost half of the products in the Desserts & Ice Cream category. Co-occurrence analyses showed that diphosphates (E450) and triphosphates (E451) were not only the most frequently used combination, but this combination also appeared more often than any single ETS appeared alone in a product.

One important finding in our study is that approximately 60% of the food products with ETSs contain more than one ETS. Multiple ETSs are notably prevalent in certain food groups, such as Bakery, Cakes & Pastries and Desserts & Ice Cream. Despite this widespread co-occurrence, to our knowledge, no studies have explored the potential health effects arising from interactions between these additives. This represents a significant gap in the literature, as co-occurrence may influence their biological effects. Moreover, because ETSs are present across diverse food groups, typical diets may expose individuals to a complex mixture of these substances – a dimension that remains poorly understood.

Carrageenan (E407), carboxymethyl cellulose (E466), and polysorbate 80 (E433) have received increased attention in recent years due to studies suggesting potential negative health effects, see introduction (56–62). In Norway, concerns about carrageenan (E407) have even prompted the food industry to remove this additive from many of their products. In our dataset, carrageenan (E407) was found in 3% of all food products (1,513 products) and in 32% of products in the Desserts & Ice cream food group (451 products). However, most food products with carrageenan (E407) were in the food group Meat & Poultry, but this makes up only 5% of the food products in this group. Carboxymethyl cellulose (E466) was found in 1.5% of all food products (707 products) and in 15% of products of Desserts & Ice cream. Carboxymethyl cellulose (E466) was excluded from the co-occurrence plot because it appears alongside a wide variety of other ETSs, and combinations found in fewer than 50 products were not included in the plot. Polysorbate 80 (E433) was only found in 24 products, making out about 0.05% of the food products in our dataset, distributed across several food groups. This may indicate that the intake of this additive contributes minimally to overall human exposure and is therefore likely to be of limited health relevance for most consumers, despite being a focus in many studies investigating its potential impact on gut health and inflammation. This highlights the importance of understanding which additives are most used in the food supply, as it helps prioritize research efforts and regulatory focus toward substances with the greatest potential impact on public health.

Intake of phosphate additives (E450–E452) and derivatives of fatty acids (E471 and E472) are suggested health risks, though less studied (63, 74). Our results show that ETSs such as diphosphates (E450), triphosphates (E451), and fatty acid derivatives (E471 and E472) are among the most widely used additives in the Norwegian food supply. These four ETSs were found across a broad variety of food groups, indicating widespread exposure through a typical diet.

The context-specific nature of our mapping of additives in the Norwegian food market makes comparison with other countries challenging. Chazelas and colleagues (66) mapped the distribution of all food additives in products on the French market in 2020, and their results were somewhat comparable to the results of the present study. The three most abundant ETSs in the French market study were xanthan gum (E415), diphosphates (E450), and pectins (E440), while the present study found that mono- and diglycerides of fatty acids (E471), diphosphates (E450), and guar gum (E412) were the top three most prevalent ETSs on the Norwegian market. Another study from the UK mapped the presence of emulsifiers across food products classified as UPFs, according to the NOVA-4 classification, in the UK Food Supply in 2023 (24). Although their study used a different categorization system, the most used ETSs in their dataset were mono- and diglycerides of fatty acids (E471), diphosphates (E450), and xanthan gum (E415). This aligns with our findings.

The presence of one or more ETS is considered an indicator that a product may be classified as UPF, according to the NOVA-4 classification system developed by Monteiro et al. (25). In general, foods that comply with the UPF definition have been shown to have a less healthy nutrient profile than less processed foods, taking factors such as energy (kJ), saturated fat, sugars, sodium, fiber, and protein into account (18). Our analysis of nutrient content across food products with increasing numbers of ETSs indicates a similar trend for energy, fats, sugars, protein, and sodium individually. However, Spearman rank correlations were weak (*ρ* = 0.09–0.24), probably reflecting heterogeneity between food groups and the fact that nutrient content is influenced by other factors such as food group, brand-specific formulation, or the intended function of the additives.

Using the same definitions of hyper-palatability, Sutton et al. and Demeke et al. found that in 2018, 69% of 6,081 analyzed food products in the US food system were hyper-palatable, an increase from around 50% in 1988 (75, 76). These results are slightly higher than the results from our analysis, where 58% (of 41,899) of the included food products met at least one of the three criteria of hyper-palatability. As some hyper-palatable foods are associated with higher energy-intakes (20, 21), this could have significant implications for public health, potentially contributing to increased rates of obesity and related metabolic disorders. The prevalence of overweight and obesity in Norway is rising (77), highlighting the need for further research to better understand the health impact of hyper-palatable foods. In general, ETSs were found widespread across all hyper-palatable criteria within the food groups. When examining specific food groups – such as Bakery, Cakes & Pastries, and Desserts & Ice Cream – products that met the Fat + Sugar hyper-palatable criterion tended to have a higher proportion of items containing multiple ETSs. In contrast, within the Snacks, Chocolate & Sweets category, a slightly higher proportion of products with multiple ETSs was observed among those that did not meet any hyper-palatable criteria. These findings partially support our hypothesis that ETSs are not randomly distributed but tend to co-occur with hyper-palatable characteristics – however, this pattern was only observed in specific food groups, such as Bakery, Cakes & Pastries and Desserts & Ice Cream.

Our results further demonstrate that ETSs more commonly appear in combination rather than individually. This observation is significant because the majority of mechanistic studies focus on single ETSs, leaving a gap in our understanding of how these substances may interact synergistically or antagonistically within biological systems. Notably, certain ETSs, such as xanthan gum (E415) and guar gum (E412), which are commonly found together, exhibit contrasting effects on colitis symptoms in mice. Specifically, guar gum has been shown to slightly alleviate colitis symptoms, whereas xanthan gum increases the abundance of a bacterium positively correlated with colitis symptoms (78). This pair, which is known to have synergistic effects on viscoelasticity (79), was also significantly enriched in several food groups, with a fold enrichment of about nine in Fish & Shellfish, and it was frequently found in combination with a broad range of other additives. Beyond this pair, our analyses further revealed that there are differences in formulation complexity across food groups. Some food groups, such as Desserts & Ice Cream, and Bakery, Cakes & Pastries, have enriched combinations of up to four additives, as well as a broad range of combinations enriched, while others, such as Snacks, Chocolate & Sweets and Meat & Poultry, were limited to both a few combinations, and only pairs. The magnitude of enrichment also varied (Fig. 10), with one of the most enriched being the three-way combination E40x & E440 & E45x, involving alginic acid and its salts (E400–E404), pectin (E440), and phosphates (E450–E452). Some combinations were rare (e.g. phosphates and fatty acid derivatives), which likely reflect segmented functional needs (some subgroups of products have different specific needs), substitutability (adding one makes another unnecessary), or incompatibility. The finding that additives co-occur systematically is hardly surprising, as additives address systematically covarying properties of foodstuffs that consumers and manufacturers wish to modify. But our study confirms the critical importance of studying the combined effects of food additives to fully understand their potential health implications.

This study provides a comprehensive overview of the presence of food ETSs across food products available on the Norwegian market, examining different food groups, nutrient profiles, and their co-occurrence. However, several limitations should be acknowledged. Firstly, the dataset utilized does not specify the proportion of the Norwegian market it represents, which may limit the generalizability of the findings. Nonetheless, the dataset includes quite comprehensive information from all major grocery stores in Norway, suggesting that it likely represents the Norwegian food market adequately and the distribution of ETSs in available food products.

While Norway is a country with under 6 million habitants (80), we believe this study serves a relevant case study to be broadened to other countries. Norwegian adults consume a high proportion of UPFs, defined according to the NOVA-4 classification, which contribute to nearly 48% of total energy intake (5). This is slightly lower than levels reported in the UK (57%) (6) and the US (58%) (12), but higher than in many European countries, where Mertens et al. report energy contributions from UPFs ranging from 14 to 44% (4). These comparisons suggest that the Norwegian food environment reflects Western dietary trends while also representing a relatively high intake of UPF-context within Europe. Furthermore, Norway’s participation in the European Economic Area (EEA) ensures that food additive regulations are harmonized with EU legislations, increasing the relevance of our findings beyond the national context.

The two databases that were used in this study used different food classification systems, leading to that similar products may have been assigned to different food groups, which could affect how accurately the groups reflect the true distribution of products. By not requiring each product to be individually reclassified, we were able to include 45,851 products in the analysis. Duplicate food products were removed using either the products’ GTIN or product name. However, otherwise identical products may have been registered under different names or GTINs, potentially resulting in duplicates remaining in the dataset. Conversely, removing products based solely on similar names could exclude items that genuinely differ in ETS content – as observed in cases where ETS content varied depending on whether products were sold whole or pre-cut.

Another key limitation of this study is the lack of information on the concentrations of additives in the food products analyzed. This gap hinders the interpretation of the findings in relation to potential health implications as clinical effects are likely affected by the amounts of these additives ingested. Moreover, the availability of products in the market may not accurately reflect actual consumption patterns, further limiting the generalizability of the study’s implications. Additionally, additive usage patterns may change rapidly due to evolving regulations or consumer pressure. For example, recent EU updates (Regulation (EU) 2025/666) (81) have revised specifications for celluloses (E460–E469), including their phasing out in foods for special medical purposes for infants and young children. In Norway, carrageenan (E407) has been voluntarily phased out by some manufacturers in response to consumer concerns. This highlights the need for continuous monitoring of additive use in food products.

## Conclusion

Our findings reveal that over one-third of the food products analyzed contain at least one ETS, and more than half meet the criteria for hyper-palatability. Additionally, the widespread co-occurrences of ETSs underscores the need for further research on the biological relevance of combinations of these food additives. In line with our expectations, these findings show that ETSs are not randomly distributed across food products, as they tend to co-occur in specific food groups. Hyper-palatable foods are especially prone to have many ETSs in Bakery, Cakes & Pastries, Desserts & Ice Cream, Meat & Poultry, and Premade Food & Dinner Kits, but not in other food groups. This indicates that the relationship between ETSs and hyper-palatability may be context dependent. As the consumption of highly processed foods continues to rise, understanding the role of ETSs in modern diets is essential for evaluating their impact on health and nutrition, and for guiding public health policy and consumer decision-making.
